# *Are teachers “butter lamps”?* Teacher stress in Chinese Minzu “ethnic” schools

**DOI:** 10.3389/fpsyg.2023.1091743

**Published:** 2023-08-22

**Authors:** Bilige Sude, Fred Dervin, Mei Yuan, Ning Chen

**Affiliations:** ^1^ School of Education, Minzu University of China, Beijing, China; ^2^ Faculty of Educational Sciences, University of Helsinki, Helsinki, Finland; ^3^ Department of Animation, Tianjin Academy of Fine Arts, Tianjin, China

**Keywords:** Minzu education, teacher stress, Inner Mongolia, case study, intercultural

## Abstract

China is a unified multi-ethnic country. Although research on teacher stress is plentiful in this context, the specific case of Minzu (“ethnic”) teachers is under-researched. Using Minzu schools located in a diverse county of the Inner Mongolia Autonomous Region (North China), as a case study, the authors examine factors behind Minzu teacher stress. Using mixed methods, three main results were identified by analyzing interviews and questionnaires with both the software NVivo and dialogical discourse analysis: societal, school and Self factors. In general, the teachers confirmed that their job was extremely stressful. While some of these aspects derived directly from the specific context of Minzu education (e.g., rural and pastoralist families send their children to towns leading to teachers taking over parents’ responsibilities), some elements were also found in other contexts of Chinese education and/or internationally. The article ends with some recommendations specifically about Minzu aspects in the treatment of teacher stress, which will also be of interest for international audiences.

## Introduction

Sayings about teachers are omnipresent around the world and often reveal, in a sense, how the work of the teacher is perceived in a given context. In Chinese, “老师是蜡烛，燃烧自己，照亮他人” translates as follows: “The teacher is like a candle, burning themselves out to illuminate others.” In one of the other languages of China, Mongolian, a language spoken by around 6 million Chinese nationals, the same saying is used, with the word “candle” substituted by “butter lamp”—a lamp that burns clarified yak butter. In both cases what the saying indicates is that teachers are seen as devoted and as having to sacrifice themselves to educate their students. Interestingly, research on teachers in China consistently emphasize that anxiety and stress are serious issues at all levels of the curriculum (e.g., Li and Zhang, 2019). However most studies tend to “patch together” Chinese teachers in a somewhat homogeneous category. In order to offer a more fine-grained picture of the “butter lamp” metaphor, this article, based on mixed methods, deals with teacher stress in China, using the very specific but underexplored context of Minzu jiaoyu (民族教育), i.e., Chinese Minzu (“minority”) education (see Dervin and Yuan, 2023), as a case study. Teachers in Minzu schools have to work with diverse and multilingual children, which, we hypothesize, might lead to different factors behind stress compared to other educational contexts in China.

At the end of 2017, there were 9.49 million full-time teachers in Chinese basic education, among which about 950,000 were full-time Minzu teachers, mostly concentrated in Chinese Minzu regions (Ministry of Education of the People’s Republic of China, 2017). China is a Multi-Minzu country, with 106 million of her citizens representing 55 Minzu “minority” groups (Rong, 2017, p. 130; examples of such groups mentioned in what follows include: Hui, Kazakh, Mongolian, Uygur Minzu groups). In this article, Minzu (民族) refers to a context of education in a specific Chinese Minzu area where teaching takes place in two languages and is targeted at Chinese Mongolian children. The area under study is called the East Ujimqin Banner (东乌珠穆沁旗; pinyin: Dong Wuzhumuqin; Mongolian: 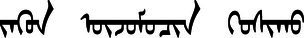), an administrative division of the Inner Mongolia Autonomous Region (Northeast of China, total area: 54,545 km^2^ with a population of 60,000, of which 74% belong to the Mongolian Minzu group). In China, Minzu autonomous regions represent almost 64% of China’s total territory and are mostly located in border regions. Multilingual education represents an important aspect of Minzu education in Inner Mongolia and other parts of the country (see Sude et al., 2020).

In this paper we focus on Minzu teacher stress using Mongolian Minzu schools in the East Ujimqin Banner as a case study. This Banner is a sparsely populated county (1.1 inhabitant/km^2^), which features a cold semi-arid climate. The choice of this Banner is based on its long tradition of Mongolian education, which provides a new and original context for studying teacher stress in Mainland China. Deep and long-term research and teacher professional development connections between the Chinese authors, who are based at a major Minzu university in China, and the schools under review also represent a motivation for undertaking this important study.

Based on the analysis of Minzu teachers’ interviews and questionnaires, the paper provides some answers to the following questions:

– What kind of stress do teachers face in Minzu schools in the East Ujimqin Banner?
– What is the role of the school and the surrounding society in Minzu teacher stress?
– What appears to be specific to Minzu teacher stress in the Chinese context?

### Problematising teacher stress

Over the past two decades, the number of studies of occupational stress in the teaching profession has dramatically increased, reflecting a growing international concern over this topic (e.g., Galanakis et al., 2020 in Greece). In general, research has shown that the teaching profession is associated with high levels of perceived stress in different parts of the world (Skaalvik and Skaalvik, 2010). As society’s expectations of children’s success increase, teachers have become a prominent group suffering from occupational stress.

The stress experienced by teachers relates to different factors. Usually the mismatch between teachers’ coping strategies and the demands of teaching leads to stress: disciplinary problems in class, unmotivated students, workload demands, reforms and changes, too many administrative tasks and poor working conditions (Yin et al., 2020).

In recent years, several studies by Chinese scholars have reached similar conclusions. Studies by Li and Zhang (2019) as well as found that teachers experience occupational stress to varying degrees. To some extent, it is inevitable for teachers to suffer from stress considering the complexity of their work (Xu, 2003). Along with individual factors, gender, school location, position, duties, education background, and teaching age determine differences in teacher stress in school and society (Wang et al., 2004). The Department of Social Medicine, School of Public Health at China Medical University proposed the following predictors for stress: chronic disease, days of sick leave, recent experience of a stressful life event and marital status. Being a class teacher was the strongest indicator of strain in the Chinese context.

As teachers are direct sufferers of stress, it has, first and foremost, a negative impact on teachers themselves. Many studies show that this negative effect is manifested in three aspects: *psychology, physiology* and *behavior*. First, teachers stress leads to negative emotions and ill-being. For instance, according to Bernotaite and Malinauskiene (2017), 34.9% of school teachers experience high levels of emotional exhaustion in the European country of Lithuania. Second, teacher stress can lead to physical illness. Malik and Björkvist (2018) have shown that long-term occupational stress can lead to musculoskeletal problems among university teachers in Finland and Pakistan. These include severe to minor health issues related to the locomotor apparatus (muscles, skeleton, ligaments). Third, teacher stress can lead to changes in behaviors. On the one hand, the occupational stress of teachers can trigger an increase in negative behaviors, which are mainly reflected in impulsive behaviors, a quick temper, reduced interest and enthusiasm (Travers, 2017). On the other hand, the occupational stress of teachers may lead to some adverse reactions to work, including absenteeism, requests for transfer or early retirement, and career changes (Ryan et al., 2017).

It is important to note that teacher stress has also a direct or indirect negative impact on students, schools and society (e.g., Huang and Liu, 2006). As far as students are concerned, teachers’ mental and physical health can also affect the quality of their instruction and, in turn, students’ motivation and learning (Gray et al., 2017). Teachers’ exhaustion and cognitive impairment caused by stress directly lead to a decline in school teaching quality.

As aforementioned, research studies on teacher stress are numerous in different contexts. However, there is a gap in the literature with respect to the applicability of this research to specific diverse groups, in particular those involving Minzu teachers in China. Most research on teacher stress and “ethnic minorities” in China, published in English, focus on the situation in Hong Kong, where the concept of Minzu does not apply since a different classificatory system in terms of “ethnicity” and “race” is in use in the Special Administrative Region (e.g., Ng et al., 2020). Yulong et al.’s (2016) study, which looks into teacher occupational strain and work ability in China among migrating Chinese Han and Hui Minzu teachers, versus Chinese Kazakh and Uygur Minzu teachers in the Xinjiang Autonomous Region of China, represents an exception, although the paper does not focus on Minzu education but on mainstream education in Chinese. They note that Han and Hui teachers experience higher levels of strain compared with the local Kazakh and Uygur teachers. According to this study, role ambiguity, responsibility and a lack of social support represent the major impediments to proper work ability.

The present paper intends to explore the main sources and characteristics of stress for Minzu Mongolian teachers working exclusively in Minzu schools in a remote area of China.

## Methods

In order to facilitate and build a more comprehensive understanding of teacher stress in a specific Minzu region of China, the paper makes use of mixed methods, integrating rigorous qualitative (semi-structured interviews) and quantitative (questionnaires) methods while drawing on the strengths and combination of each (see Poh, 2023). Integration of the qualitative and quantitative research activities was negotiated between us in relation to the aforementioned research questions and occurred during data collection, analysis and presentation of results. Contents of the different data and analyses were compared and related, while discussions of areas of convergence and divergence took place between us in order to provide general interpretation of the entire data set. Since there is no consensus on the philosophical foundations of mixed methods research, we adopted a pragmatist position whose tenets are described by Shan (2022, p. 3) as follows: “both the mind independent physical world and the constructed social and psychological worked exist, and the reality is complex and multiple (…), social scientific research is value-oriented (…) and the aim of social scientific research is to solve problems (…).” To us, this means that the careful choice of a mix of methods, data and procedures of research can support obtaining a variety of perspectives on the important issue of teacher stress in a Minzu context. This can also provide a robust description and interpretation of the data, reinforcing the understandability of quantitative results and applicability of qualitative findings.

### Research site

The East Ujimqin Banner is a Chinese Mongolian Minzu community with a concentrated Mongolian population. A total of 20 schools are to be found in the Banner: two secondary schools (including a vocational school), five primary schools and 13 kindergartens. A total of 7,195 students attend the schools, including 2,870 middle school students and 4,325 primary school students (People’s Government of East Ujimqin Banner).^1^ A total of 848 primary and secondary schoolteachers work in the Banner, among which 510 are from different Minzu groups, accounting for 60% of the total number of local schoolteachers. We have selected five out of the seven Minzu primary and secondary schools in the area to study teacher stress. The choice of the schools was based on both their strong Mongolian Minzu identity and long-standing cooperation with the institution of the Chinese authors of this article, especially in terms of research and teacher professional development.

### Participants

A total of 50 Minzu primary and secondary schoolteachers took part in the semi-guided interviews. Thirty seven participants were females and 13 males; 42 participants were employed full-time and eight worked part-time. Their average age was 38 years, with an average of 8 years of teaching experience (with a minimum and maximum of 1.5 and 28 years). Besides the interviews, 100 teachers of all subjects in all grades in the participating Minzu primary and secondary schools were randomly invited to participate in the study by completing a questionnaire on paper. Eighty one were females and 19 were males; 90 participants were employed full-time and 10 worked part-time. Their average age was 36 years and their average work experience as teachers was 7 years (with a minimum and maximum of 1 and 30 years, respectively). All in all, the participants represent a good mix of different Minzu groups (Mongolian/Han), subjects, grades and experiences.

### Ethics statement

The participants were identified in cooperation with the leadership of the five schools, who sent a recruitment message in both Chinese and Mongolian to all their teachers several months before our visits to the schools. The message introduced the Chinese and Finnish teams, their research interests and the objectives and practicalities of the study. The message insisted on the voluntary aspect of the study and introduced the international standards of ethics followed by the research team, informed both by the Finnish National Board of Research Integrity (since two scholars (Dervin, Chen) from Finland were included) and ethical guidelines of the Chinese Educational Research Association. For example, the recipients of the message were told that all the interview data would be collected in the privacy of a quiet office at the school during working time and that they could retract from the study at any time.

Volunteers contacted back principals to express their interest in being either interviewed or filling in a questionnaire. Following the study, two members of the research team went back to the school to introduce the most important findings to all the teachers and school leaders as part of a professional development course. No obvious problems related to ethics were noted before, during and after conducting the study. Since our approach to research on issues of diversity and interculturality pays specific attention to multilingual aspects, which are an integral part of research ethics, we spent quality time together observing, discussing and renegotiating the ways we translated the Chinese excerpts used in the following analysis (Dervin, 2023) to ensure transparency and doing justice to what the participants uttered during the study.

### Tools and methods

Although the research documents were produced in both Chinese and Mongolian, the data were collected in Chinese and translated into English for the analysis. Eight research assistants supervised by the research team interviewed teachers in the participating schools in the Banner and collected the questionnaires on the spot. Two researchers spoke Mongolian and could help if communication in Chinese was deemed to be a problem. However, since the teachers were all fully bilingual (Chinese/Mongolian) Mongolian was thus not required.

The empirical materials comprise of semi-guided interviews (duration between 30 min and 1 h) and questionnaires. The same 10 questions were asked both during the interviews and in the questionnaires and dealt with *teachers’ daily lives, classroom experiences, communication within and outside the school with students and parents, need for further training, Minzu aspects of teaching-learning*. We argue that the use of questionnaires was important to add elements to the data collected during face-to-face interviews. As mentioned earlier, the topic of teacher stress in such a diverse context as Mongolian “Minzu” schools is under-researched and we felt that having access to both interviews and questionnaires would help us identify more perspectives on the topic.

The questions to be used for the semi-guided interviews and questionnaires were pretested in order to evaluate potential problems for both interviewers and participants and to improve data quality so that we could meet our research objectives better (Presser et al., 2004). This is why we made use of the cognitive interview method, “focusing on providing a view of the processes elicited by the questions” (Presser et al., 2004, p. 111), to pre-test the questions. Five Mongolian student teachers from the Chinese scholars’ institution (at Master’s level), who had some experience in the Mongolian Banner under question, were thus presented with the questions for both interviews and questionnaires in Chinese and asked to share their thoughts on them in interaction with us (“think-aloud”). A few problems were diagnosed, rectified and rediscussed with the student teachers. For instance, the question concerning specific Minzu aspects of teaching-learning was found to be too direct and somewhat ambiguous in the way it was formulated since the majority of student teachers argued that most teachers in the Banner do not receive a specific “Mongolian” Minzu perspective during their teacher education and training (a fact that was later confirmed in discussions with teachers and leaders in the schools). The student teachers suggested instead to start with a broader question about problems encountered in teaching-learning and to follow it up by a subquestion that includes the word “Mongolian” rather than “Minzu.”

The entire data processing and analysis was done first by means of the software NVivo (NVivo 11, 2015). NVivo has the capacity to help researchers organize, analyze and find trends and insights in, e.g., interviews and questionnaires by classifying, sorting and arranging the information found in them. By creating projects, collecting source materials, creating nodes, coding meanings and highlighting key points, the results can be presented in the form of reports or tree-structure diagrams (McNiff, 2016). Through subject coding and hierarchical coding of interview materials and questionnaires, we analyzed the main characteristics and sources of stress of Minzu primary and secondary school teachers.

In the following analysis, dialogical discourse analysis is also used as a complement to examine the excerpts that we have carefully selected to illustrate different aspects of teacher stress in a Minzu school context (Grossen, 2010). Dialogical discourse analysis has been amply used in research on diversity in education and beyond (Simpson, 2022) in order to explore further the complexities and instabilities of the topic. In analyzing the data, we paid attention to the concrete voices that the teachers included in the interviews and questionnaires, as well as their reaction to these voices (Bakhtin, 1981). Such voices are identified in the teachers’ use of for instance personal pronouns (*you, they*), the passive voice (as in *I was told to do this, told* indicates that someone else is talking), the inclusion of modal verbs and adverbs (as in You *should not say* this; *should* also hints at the presence of someone’s voice).

#### Analysis: teacher stress in Minzu schools in a Chinese Mongolian Banner

**Figure 1:**
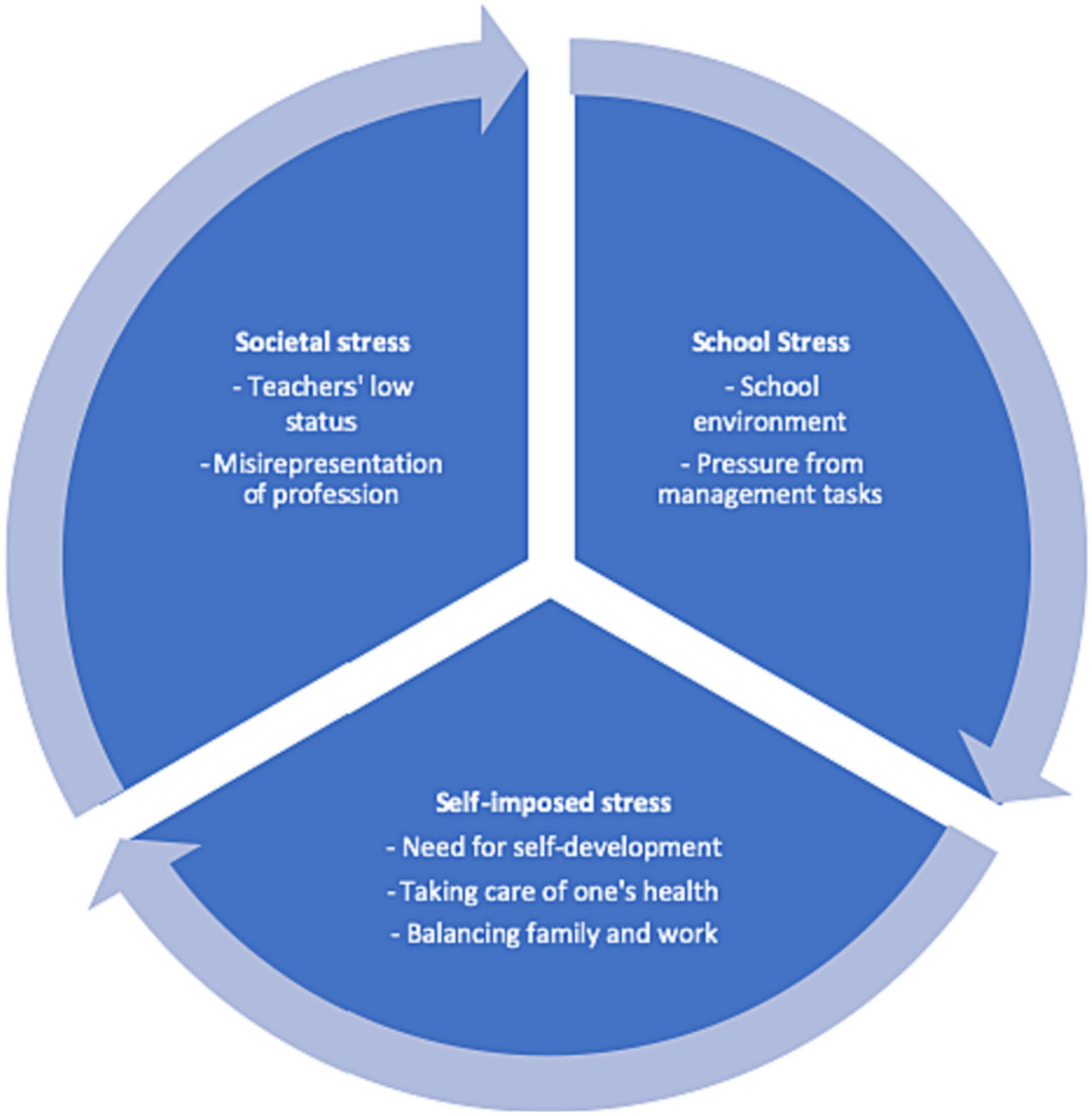
Summary of teacher stress in a Minzu school context.

Through coding trends and insights identified in both interviews and questionnaires by NVivo (Tables 1, 2), it can be concluded that, based on the data, teacher stress in the five schools shows similarities, mainly from three aspects: *society*, *school* and *self*. As can be seen in the subtopics found in the two tables, *Stress from Society* relates to the teachers’ low social status; misunderstanding of the occupation of teachers from the broader society. *Stress from School* has to do with dissatisfaction with the school setting and environment; heavy teaching load; and complex management tasks. *Stress from Self* includes the driving force of self-development and balance between family life and work. Figure 1 summarizes the main three findings.

**Table 1:** Nodes, coding references and items coded.

Nodes	Number of coding references	Aggregate number of coding references	Number of items coded	Aggregate number of items coded
Node\\Stress from society\Low social status	38	38	20	20
Node\\Stress from society\Misunderstanding teacher profession	30	30	14	14
Node\\Stress from society\Imperfect public service	33	33	16	16
Node\\Stress from school\School setting environment	95	95	51	51
Node\\Stress from school\Heavy teaching tasks	83	83	39	39
Node\\Stress from school\Complex management tasks	35	35	17	17
Node\\Stress from self\The driving force of self-development	66	66	37	37
Node\\Stress from self\Balance family and work	59	59	38	38
Node\\Stress from self\Focus on own health	27	27	15	15

**Table 2:** Trends and insights from the interviews and questionnaires.

Stress from school		Stress from self	
School setting environment	Teaching stress	Driving force of self-development	Family
Poor working condition (salary), cumbersome tasks, office environment, holiday, material welfare, home-school communication, school leader	Academic achievement	External driving force (professional title, authority)	Economic pressure
	Bilingual teaching	Internal driving force (teacher professional growth)	Family-work balance
Stress management		Focus on own health	
		Stress from society	
Head teacher, daily behavioral management, Minzu students		Misunderstanding the teaching profession, social status, social public service	

Due to space limitation in the paper, 12 carefully selected and representative excerpts from both interviews and questionnaires are introduced and analyzed to illustrate the three dimensions in what follows.

#### Societal stress: socio-economic stress and misrepresentation of the teaching profession

##### Low socio-economic status of teachers leads to a lack of respect

Chinese society is often said to be proud of its long-standing tradition of respecting teachers (Gu, 2014, p. xii). This tradition can be traced back to the time of the philosopher and educator Confucius (591–479BC), who devoted his life to teaching and education in the hope of restoring social and moral harmony. However, dramatic and sometimes turbulent changes in the country’s political, economic and socio-cultural environments over the past more than seven decades have posed profound challenges to the traditional image of teachers and, more importantly, how teachers themselves perceive their professional identity, commitment and effectiveness in light of these changes (Gu, 2014).

According to the data, this is also the case for teachers in Minzu areas. In recent years, although the status of teachers in Minzu areas has improved, it is still at a low level. This can be clearly seen from the questionnaires and interviews derived from a specific Minzu area. According to our analysis of the entire data, up to 57% of the participating teachers believe that they have a low social status; 21% share the opinion that they have an ordinary social status; and another 7% believe that they have a high social status. In the interviews, most teachers talked about their social status and considered it to be an important source of professional stress. This is the case of Teacher B, who had been teaching for 10 years in the Banner:

Excerpt 1

The status of teachers is at a low level. We don’t feel that there is much respect for teachers in society in everyday life. We are just like ordinary people in other professions. Anyway, we don’t feel anything special about our social status. To put it bluntly, we are working for schools and we are serving others.

In her direct answer to the question “How do people perceive you as a teacher in your community?,” Teacher B uses the generic pronoun “we” which seems to encompass “society” and/or “teachers” as a way of supporting her answer (Grossen, 2010). Negative sentences also fill her answer: “we do not feel much respect for teachers” (“society”) and “we do not feel anything special” (“teachers”). The end of the first excerpt seems to summarize the main argument of the teacher through the use of the word “serving” (服务 in Chinese, also means “to give service to”), which, at first, may have the negative connotation of “lack of agency.” We note, however, that in Chinese, the word hints at the second category of public services as defined in the Jacques Payne Public Service Classification: social and cultural services, including education, health, social security, and cultural activities (Chen, 2007). After getting a teaching job, Chinese teachers may apply for the status of “civil servant” (有编制), which gives them many social and financial benefits. The central government’s transfer funds are mainly used in the central and western regions of China, more precisely in Minzu areas, border areas, poverty-stricken areas, major grain-producing areas, mineral resources development areas, and areas with challenging ecological protection tasks so that infrastructures as well as education, health, culture and other public service facilities can be improved (Tang, 2018). In that sense, teachers have the responsibility of “serving” to improve the future socio-economic position of students. This seems to produce a lot of pressure on teachers but does not lead to a strong positive social position.

In the questionnaires, we also found that some teachers misunderstood the relationship between *social status* and *economic performance*, and that social status seems to be perceived as positively related to income: The higher the income, the greater the social status. For example, Teacher C (5 year-experience) asserted: *“here, the status of teachers is not high because our wages are low. Can low wages symbolize high status?.”* This may be stressful in that teachers think that social status exerts pressure on them. One reason is what some of the teachers refer to as “economic” stress. It is important to note here that Minzu teachers get the same salary as “ordinary” teachers in other schools and that, in China, teachers’ salaries are decided upon at the local level, based on the economic conditions of the region.

##### Misunderstanding the teaching profession: what does a teacher do?

An important factor that emerged from the interviews was the lack of understanding of the teaching profession by the surrounding Minzu society, which adds to teacher stress. Moreover, this seems to emanate from a deficit view of teachers. For Dinishak (2016) deficit approaches conceptualize “the target individual or group primarily (or even solely) in terms of their perceived deficiencies, dysfunctions, problems, needs, and limitations.” For the teachers in our study, this often results in an increased workload, and failing to acknowledge the importance of their wellbeing and commitment.

The first stressor for the teachers is Minzu parents’ misunderstanding of teaching as an occupation. Teacher C (3-year work experience) explains during the interview:

Excerpt 2

Some parents now don’t understand the teacher at all. They think that the teacher is responsible for everything to do with their children. As long as children are sent to the school, there are no parents. Counselling and care for the child are not enough. The teacher is given full responsibility. If his child’s grades are not good, they will blame the teacher.

Teacher C seems to make a clear link between the teachers’ perceived responsibilities (among others: “counseling,” “care,” “good grades”) and some of the Minzu parents’ passive yet somewhat aggressive attitudes toward them (“they will blame the teacher”). The repetition of the words *responsible/responsibility* (accompanied with “full” and “for everything”) seems to emphasize the pressure felt by the teacher from the parents. In a similar vein, Teacher D (12 years of experience) uses emotionally tainted words to describe some of the parents’ attitudes toward them (“too disrespectful,” “simply hate the teacher,” “distressing”) in their answer to the questionnaire:

Excerpt 3

Now some parents are really too disrespectful. They don’t understand the hard work of the teacher. They give us a problem. The children have problems. Some parents don’t pick up any phone calls. Otherwise, they communicate directly with us by phone. They say that my child is not a learning material, and does not need a teacher. Some parents say that it is the responsibility of the teacher and blame the teacher for everything. Others simply hate the teacher and feel that it is easier to be a teacher and arrange homework. If we let parents sign off children’s homework, they think the teacher is shirking in their job, and it is the teacher’s job to let the parents do it. It’s really distressing to meet these parents.

Teacher D uses many represented (generic) voices of Minzu parents in this excerpt as if he was authentifying their voices (“they say…”; “some parents say…”) (Dervin, 2023). Similar arguments were identified in many interviews too: some Minzu parents mistakenly believe that teachers’ work is easy and thus they do not have compassion for teachers; other Minzu parents do not wish to cooperate with teachers (e.g., homework) and either let them do all the work or blame them for any problem encountered by their children. It is important to note that some Minzu parents do not seem to understand the value of having their children educated (see in questionnaire excerpt 3: “my child (…) does not need a teacher”).

In addition to parents, the school leadership is presented as having a limited understanding of and care for teachers. In excerpt 4 based on an interview, Teacher B (10-year experience) shares her frustration with the extra stress imposed by leaders:

Excerpt 4

Leaders sometimes don’t understand the actual situation. For the school’s performance and rankings, for our teachers, the stress on students is too much. Let us focus on teaching. We also need to deal with the various inspection tasks that leaders impose and arrange temporarily. With limited energy, the amount of working time left to control the children is limited.

The gap between the work of Minzu teachers and the pressure from the leaders is well described in the excerpt through alternating between what the job should be about (“teaching,” “control the children”) and what the leadership imposes on teachers (“performance and rankings,” “inspection tasks”). These problems seem to be generally shared by teachers in other parts of China but also in the rest of the world (see, e.g., Lambersky, 2016 in Canada).

Another major source of stress, as reported by teachers, was their workload beyond teaching, of which many of the aforementioned actors (e.g., parents, school leaders) do not seem to be aware. Lazarus (1990) defines stress as an individual’s realization that environmental and personal demands have exceeded the capacity of their coping resources. All the teachers reported that many non-instructional workload issues, such as school meetings, parent evenings, students’ behavior, keeping up with the use of technology and staff professional development, increased their level of stress (see also Sass et al., 2011). According to the participants, parents and school leaders who have the most contact with Minzu teachers do realize the importance and value of teachers’ professional work. However they also add to their responsibility and accountability by exceeding the capacity of their coping resources. In general the understanding of the teaching profession in this Minzu context is “broad” in the sense that it is perceived as going beyond “mere” teaching. This seems to lead the teachers to feel they lack the support that they deserve.

#### Stress related to the school: the environment and (non-)teaching obligations as factors

##### The school environment

According to Saeki et al. (2018) teachers develop through continuous interaction with the school environment. As such a healthy school environment is an important condition to support and guarantee the development of teachers. The effects of the school environment can be intense. According to Li and Dervin (2018), the school environment includes the physical environment of the school, the school’s interpersonal environment, home-school cooperation environment, professional development environment and classroom teaching environment, as well the school’s socio-cultural environment. Teachers’ perceived stress can also result from a relative lack of balance between demands and resources at school. In our study, the stress from the multifaceted perspectives of the school environment affecting teachers was linked to the institutional environment, classroom teaching environment, professional development environment, home-school cooperation environment and school physical environment.

The stress imposed by the classroom teaching environment is mainly due to the discrepancies between the number of teachers and students. Many teachers from different schools believe that the current number of students does not match the number of teachers. In other words, there appear to be too many students but too few teachers so the teachers seem to suffer from overload and teaching stress. This opinion is shared by Teacher E (experience of 2 years) in the questionnaire:

Excerpt 5

If you have more than 60 students in a class, what kind of students do you focus on first? You will not be able to take care of the students who have achieved good grades. And vice versa. We have major headaches.

Like most teachers in the data, Teacher E does not speak in her name but uses a generic voice (“we” and “you”) to describe the problems that overcrowded classrooms represent for her (“you will not be able to”; “we have major headaches”). What the teacher is saying here is that, when there are too many students in a classroom, she cannot take care of all the children, and thus feels that she acts unprofessionally. The stress of the professional development environment on teachers is reflected in the lack of professional training provided (to them) because of funding. Teacher training is an important way to achieve professional development, and it is also a way to improve teaching practice (Li and Dervin, 2018). Also, it is an effective way to solve some of the dilemmas that bring stress to teachers. Effective teacher training appears weak in the Banner, which has led many teachers to solve the problems in their classroom teaching and student management by themselves. This seems to increase their burden and create a sense of stress. According to our analysis of the entire data, 35.67% of the teachers did not receive any training, which led to a lack of relevant professional knowledge when they encountered difficulties in their job.

The stress from the home-school cooperation environment put on teachers is reflected in the somewhat precarious cooperation between home and school. Minzu schools in the Banner receive students from remote Mongolian rural and pastoral areas. The transfer of parental responsibilities to teachers has led to an increase in the workload of teachers. Parents from the remote areas tend to be less educated and offer little attention to and support for their children’s learning. Students board at school and only leave school when they are on vacation. Because students are in contact with teachers all day long, the latter must assume some of the responsibilities of parents. In the interviews many teachers reported that, at school, because of the long-term lack of parental care and love, students have many problems in terms of psychology, conduct and habits. This requires teachers to spend more time and energy to deal with these “extra” aspects of education, and “substituting” the parents.

Finally, the stress imposed by the physical environment of the school on teachers is reflected in the poor office environment and lack of information technology teaching aids and materials. Teacher G (8 years of experience) maintains in her interview:

Excerpt 6

Our school is a hundred-year old school… the required scores for entrance test in the town are among the top three. Although our teaching quality is quite high, the school conditions are not very good, the classrooms and offices are old, and the computers too. We can’t provide what is needed to teachers wanting to follow modern teaching methods. Hence, we always worry that one day our school will be merged with other schools.

In this excerpt one can clearly feel the stress that the poor physical environment of the Minzu school seems to trigger in the teachers. The excerpt starts with Teacher G somewhat boasting about the good quality of the school in academic terms, with a reference to its competitive entrance test and the “high quality” of teaching. However the rest of the excerpt describes and evaluates the conditions of the school: “not very good,” “old”—opposed to “modern.” Beyond the technical and practical reasons behind the stress this causes seems to lie the fear of merging the school with other schools as a consequence of the “poor” physical school environment. Since the 1990s the compulsory school merger program, whereby some schools in some areas were abolished to group together students in centralized schools, has been a constant source of stress for some educators in China (Chai et al., 2017).

##### Heavy teaching tasks: fulfilling curricular and extra-curricular obligations

Teachers have both curricular and extra-curricular obligations (Kyriacou, 2001). In the schools under review these include planning, preparing and conducting lessons, supervising playgrounds, organizing school events, liaising with administrative staff, but also communicating with parents and/or researchers. Teachers may feel worthless because they cannot achieve their personal goals or are incapable of making a difference to their students’ lives (Dworkin and Tobe, 2012). As a student’s achievement diminishes, the teacher’s sense of accomplishment weakens too, because teachers have less evidence that teaching practices are effective in promoting student learning (Hence, 2016). NVivo coding results of the data show that student achievement is the biggest influencing factor in relation to teaching stress.

In her questionnaire, Teacher F, who has 5 years of teaching experience in the same school, maintains that students’ performance is the main source of stress for her:

Excerpt 7

When the students’ performance is stable, the stress is still minimal. If the students’ performance suddenly drops, we are very nervous. I feel that I have not taught well, and I feel ashamed in front of the principal and parents, so the stress is very high.

Interestingly in this excerpt, the teacher alternates between a groupal identification (“we are very nervous”) to a reference to her own self (“I feel…” used twice) to describe her feelings toward the two main actors whom we have discussed before, and who represent a major source of stress for teachers in relation to students’ performance: leadership and parents.

The fact that there is not enough teachers and that some are requested to teach more to compensate, also seems to add to teacher stress, especially in their fear of not doing their job properly, leading to students failing. Teacher G, who is one of the most experienced teachers in the study with 17 years of classroom practice, explains during her interview:

Excerpt 8

The teaching task is very heavy now, the leader assigned me to teach English to four classes, to teach so many students. If the preparation is not enough, if there are mistakes, some students will not be able to learn properly. Therefore, it is very hard to prepare for a new class every time. It takes several days, and the task is stressful. I am afraid that students will not learn well.

In the excerpt, the word choice is indicative of the negative evaluation of this aspect of teacher stress: “it is very hard,” “the task is stressful,” “I am afraid the students will not learn well.” The fear of students’ failure triggered by the teacher’s work is a theme that permeates most of the interviews since teachers in the Banner are held accountable for this aspect by both the society (parents) and decision-makers (leadership).

##### Complex management tasks

Collie et al. (2012) argue that teacher burnout, defined here as “a syndrome of emotional exhaustion, depersonalization, and reduced personal accomplishment” (Skaalvik and Skaalvik, 2010, p. 1062), occurs in light of management tasks such as school safety, student misbehavior and changing accountability standards. While there are many causal factors of stress among teachers, stress related to student behavior and discipline, is consistently mentioned in research. Schools that have strong accountability measures and a drive for educational reform establish policies that may contribute to teacher stress and burnout (Berryhill et al., 2009). Issues that interfere with a teacher’s instructional time were also listed as the most frequent cause of stress among the Minzu teachers. These include: increasing levels of paperwork, extracurricular duties and interpersonal conflicts.

The Mongolian (minority)-Han (majority) Minzu ratio in the Banner under review is 3:2. Mongolian and Han students study in the same schools. Due to the language barrier (Mongolian/Chinese), somewhat different living habits and identity claims, disagreements among students can occur. These disagreements can lead to incidents such as bullying and fights. In his questionnaire Teacher H (teaching experience of 10 years) is the director of teaching and discipline in one of the schools:

Excerpt 9

Conflicts between students of different Minzu groups can easily become issues during this sensitive period and in this region. Our country has always attached great importance to the issue of national unity, and teachers can certainly not bear the charge of ‘undermining’ this principle. Therefore, teachers should always monitor communication and psychological states among their student groups and carry out mediation work as part of their teaching. However, mediation is not an easy task. Any punishment should be fair and just, and comprehensive schools must deal with it well. Considering the overall situation, in order to achieve an appropriate balance between students, parents, colleagues, schools and even Minzu groups, and to address the interests and demands of both sides from root causes, teachers need to make great efforts, which increases the stress on Minzu teachers. Such stress is more important for them, which makes them different from other teachers.

In this excerpt Teacher H compartmentalizes teachers into Minzu and non-Minzu (meaning: Han) teachers. As he admits to conflicts taking place at times between students of different Minzus, for him, teachers of Mongolian Minzu identity are more concerned by these clashes and thus experience more stress than other teachers. The role of these teachers in this regard is determined by Teacher H by means of a modal verb of obligation (“should”) indicating a strong ethical commitment: “teachers should always monitor… and carry out mediation.” This complex role of mediators, which seems to be taken very seriously by Mongolian Minzu teachers, relates also to the Chinese political objective of “diversity in unity”—acceptance of difference while feeling united to the Chinese Nation (see Yuan et al., 2022).

#### Stress related to Self: pressure to self-develop and balancing family and work

##### The driving force of self-development

Although Minzu teachers experience stress from society and school work, many shared their wish to self-develop.

First of all, “obtaining the civil servant status” and “promotion” are the two most frequently used phrases in the data. Teachers expect to reach the civil servant status and to get a promotion via a “self-improvement drive.” Having an officialized status as a civil servant is considered to be an important part of teachers’ professional identity in China, which gives them some security. As such teachers’ salaries are overseen by a state public utility fund and are not subject to market competition. Teachers’ wish to obtain the status but also to get promoted puts a lot of stress on them (Wang, 2005).

Second, based on our data analysis, it can be concluded that teachers’ self-efficacy is also an important factor affecting their professional stress. Following Skaalvik and Skaalvik (2010, p. 1059) we understand *self-efficacy* as people being “self-organizing, proactive, self-regulating, and self-reflecting,” which affects positively or not their goals and behaviors. By means of self-efficacy teachers can “believe in their own ability to plan, organize, and carry out activities that are required to attain given educational goals” (Skaalvik and Skaalvik, 2010, p. 1059). Personal experience, mental state, social environment, school management and interpersonal relationships as well as teacher factors such as self-evaluation can have an important impact on teachers’ self-efficacy. Teachers with high self-efficacy have confidence in their own abilities, have a strong sense of self-control, often set higher-level goals, attempt difficult work and demonstrate strong target commitments, thus accelerating their professional development. Teachers with low self-efficacy represent the opposite (Zhao and Li, 2006). Many studies show that teachers’ perceived stress is associated with self-efficacy and motivation and that it has an impact on their students’ motivation and achievement (Fernet et al., 2012). Thus, teachers who feel stressed at work and less self-efficient, teach less enthusiastically, which could have a negative impact on the quality of their lessons. It can be seen from the data that many teachers have a strong desire for self-improvement in areas such as teaching, management and inter-Minzu understanding. In her interview Teacher D (12-year teaching experience) shares her worries about the omnipresence of technology in education:

Excerpt 10

Now information technology is changing too fast, and social progress is also fast. When we were young, the consumption and use of technology by the children was not a problem. The children’s eyes are wide open now, the mobile phone is ubiquitous, we don’t improve. I will be redundant soon, and then I can’t teach. This stress is really great.

In the data, references to technology and teaching-learning systematically lead to the teachers exhibiting low levels of self-efficacy. The fear of “being (made) redundant soon” (being seen as useless) from excerpt 10 is also shared by Teacher K (teaching experience of 9 years) in her questionnaire:

Excerpt 11

Now, at the end of each lesson, teachers are required to self-evaluate, do self-reflection. The biggest problem at present is that they feel that their knowledge is far behind the speed of student progress. When we are teaching, if we teach students knowledge and they feel that we are not capable, it’s really hard for the students and they may disobey us. We have to improve quickly. Teachers’ lives are difficult now.

The use of words associated with speed (e.g., “we have to improve quickly”) seems to suggest that teachers fall behind the kind of knowledge and skills that are required to work with today’s students. From the extracts above, in order to be better qualified for education and teaching, teachers must have a sense of accomplishment in their careers. Although the concerns shared by the teachers in this section seem to be shared by teachers in other contexts (see Kiili et al., 2016), the wish to self-prepare for dealing with inter-Minzu communication and understanding in the schools was mentioned by several teachers, but it was never fully problematized, just mentioned in passing.

##### Balance between family and work

Due to the special geographical environment, demography and socio-economic characteristics of the Banner, Minzu schools are mostly concentrated in towns, which causes teachers’ families located in pastoral areas to be separated. Our analysis shows that the family is a factor that creates a lot of stress for teachers, mainly because it is difficult to balance family and work.

Teacher K (experience of 8 years) points out during his interview:

Excerpt 12

What’s uncomfortable now is the long distance. My wife and I are far away from where we work now, because it is usually the job that dictates where we are. My wife and I sometimes can’t see each other for two, three or six months, even if there is stress in our hearts, we can’t leave our jobs. Some teachers are older, their children go to school elsewhere or they live apart from their children when the children are very young, and their children live alone in a central town at primary school.

In this excerpt the teacher describes the problems that his family faces as well as the case of “older” teachers. Separation from family, in both cases, seems to represent a norm in teachers’ lives in the Banner, which adds to teacher stress.

Besides the long-term separation between husbands and wives, in the interviews for instance, more than half of the teachers talked about the lack of time spent with their family due to long working hours in school, the impossibility of taking care of children, and the inability to care for the elderly. Many teachers explained that they spend more time in school than at home with their children. In this regard, most teachers expressed their increasing sense of guilt toward their families because of their work. Through the NVivo analysis of the entire data set, we identified that the phrase “care for children or the elderly” appeared frequently in the coded results, and many teachers in the interviews talked about their helplessness regarding care of the family. It is then obvious that Minzu teachers’ family conditions have an influence on their work.

## Discussion and conclusion

In this paper, taking Minzu schools located in the East Ujimqin Banner, an administrative division of the Inner Mongolia Autonomous Region in China, as a case study, we looked into Minzu teacher stress. Three main results were identified by analyzing both interviews and questionnaires. Stress emerged mainly from three elements: societal, school and Self factors. Societal stress translated in terms of socio-economic stress and the misrepresentation of the teaching profession. Stress related to school shed a light on the influence of the school environment and of (non-)teaching obligations. Finally discourses that revealed pressure to self-develop and the difficulty to balance family and work were categorized as stress related to Self. In general, the teachers confirmed that their job was extremely stressful. Following the rigorous use of mixed methods in the paper, discussion of data collection, ethics and analytical protocols, we believe that the study can be replicated in other Minzu contexts in China.

It became clear in the analysis that stress derived directly from the specific context of Minzu education, while other aspects of teacher stress are to be found in other contexts of Chinese education and/or internationally. The influence of poor working conditions that some teachers described as well as intense administrative tasks and the lack of professional development on teacher stress have been described elsewhere (e.g., Geiger and Pivovavora, 2018 in the US). What seems to lead specifically to Minzu teacher stress includes:

– Minzu rural and pastoralist families send their children to towns in the Banner, which leads to long-term separation. Teachers have to take over the role of parents during school time.
– According to the teachers, some of the Minzu parents do not see the importance of education positively and misunderstand the role of teachers, blaming them for putting too much pressure on their children.
– The Minzu schools under review are also inter-Minzu schools since members of different Chinese Minzu groups study in the same schools. At times, inter-Minzu disagreements and conflicts might happen, which adds to Mongolian Minzu teacher stress. As a consequence, teachers feel the need to self-prepare for inter-Minzu communication and understanding, although they do not seem to know how to do it and have not received specific training during their pre-service teacher education.

In order to conclude this article we would like to make some recommendations specifically about Minzu aspects in the treatment of teacher stress. Although these elements might apply particularly to the Chinese context under review, we believe that other contexts of “inter-ethnic contact” and multiculturalism might also consider these recommendations.

At the national level, it is increasingly important to strengthen policies concerning the work of Minzu teachers. There is a need to improve their social status by making them feel more confident about their work and to alleviate some of their burdens, worries and anxiety. For example, ensuring targeted development of Minzu language resources, including digital teaching resources and book resources, might help reduce the workload of Minzu teachers. A financial incentive might also increase their motivation and sense of pride of being teachers.

The shortage of Minzu teachers leads to a sharp increase in the intensity and stress of individual teachers. Therefore, proper recruitment of Minzu teachers should be ensured. In order to improve teacher career management and promotion, a specific policy of promotion and review for Minzu teachers is necessary. Increasing the proportion of middle and senior job positions for Minzu teachers in primary and secondary schools in border areas is also *de rigueur*.

Training mechanisms for Minzu teachers should also be improved since they can affect the development of education and reduce stress by helping them develop certain coping strategies. In view of the unique requirements of Minzu education and the differences between the training of Minzu and “ordinary” teachers, it is indispensable to clarify, for example, the responsibilities and missions of Minzu teachers. In other words, Minzu teachers should be prepared to improve their professional qualities continuously. More importantly, Minzu teachers should be provided with the skills to learn to adjust and strengthen their own stress-resistant abilities.

Going back to the Mongolian “butter lamp” metaphor about teachers of the beginning of this article, we could compare it to a similar metaphor from a very different context, Finland, where teachers are often described as being “candles of the nation” (“kansan kynttilä” in Finnish), i.e., powerful, academically trained and independent figures who enlighten their students but never “burn out” (Li and Dervin, 2018). Minzu teachers could also be made to illuminate their students by burning their butter lamp, however they should not burn out by themselves but be empowered and supported to bring light continuously.

## Data availability statement

The raw data supporting the conclusions of this article will be made available by the authors, without undue reservation.

## Ethics statement

The studies involving human participants were reviewed and approved by the Minzu University of China, School of Education Ethics Committee. The patients/participants provided their written informed consent to participate in this study.

## Author contributions

BS supervised data collection, built the drafts of the manuscript, wrote the methodological and analytical sections and liaised with other authors. MY collected and supervised data, contributed in writing to theoretical framework, data analysis and discussions. NC collected and translated data, contributed in writing to analysis, introduction and discussion. FD supervised the study, supported in writing with theoretical and methodological knowledges, discussions, checked translations and proofread the manuscript at all stages. All authors contributed to the article and approved the submitted version.

## Conflict of interest

The authors declare that the research was conducted in the absence of any commercial or financial relationships that could be construed as a potential conflict of interest.

## Publisher’s note

All claims expressed in this article are solely those of the authors and do not necessarily represent those of their affiliated organizations, or those of the publisher, the editors and the reviewers. Any product that may be evaluated in this article, or claim that may be made by its manufacturer, is not guaranteed or endorsed by the publisher.

## Footnotes

^1^ http://www.dwq.gov.cn/
